# Supplementary material for: Understanding the complementarities of surface-enhanced infrared and Raman spectroscopies in CO adsorption and electrochemical reduction
Source: Nat Commun. 2022 May 12;13:2656. doi: 10.1038/s41467-022-30262-2 (PMC9098881; doi:10.1038/s41467-022-30262-2)
Supplement: Supplementary file 1 — Supplementary Information [file 41467_2022_30262_MOESM1_ESM.pdf]

Supplementary Information

Understanding the Complementarities of Surface-Enhanced Infrared and Raman Spectroscopies in CO Adsorption and Electrochemical Reduction

Xiaoxia Chang[1,2,3], Sudarshan Vijay[4], Yaran Zhao[3], Nicholas J. Oliveira[3], Karen Chan[4,*] and Bingjun Xu[1,2,3,*]

[1]College of Chemistry and Molecular Engineering, Peking University, Beijing, 100871, China
[2] Beijing National Laboratory for Molecular Sciences, Beijing, 100871, China
[3]Center for Catalytic Science and Technology, Department of Chemical and Biomolecular Engineering, University of Delaware, Newark, Delaware 19716, United States
[4]CatTheory Center, Department of Physics, Technical University of Denmark, Kongens Lyngby, Denmark 2800

* Corresponding authors: kchan@fysik.dtu.dk, b_xu@pku.edu.cn

Basic Information of IR and Raman Spectroscopies

Basic Working Principles of IR and Raman Spectroscopies. Surface-enhanced infrared absorption spectroscopy (SEIRAS) and surface-enhanced Raman spectroscopy (SERS) are two informative techniques to probe vibrational modes of adsorbate species at solid-gas and solid-liquid interfaces[1,2]. SEIRAS and SERS do not interfere with electrochemical reactions, and thus are frequently employed to identify reaction intermediates in electrocatalytic systems, which are then correlated with reaction activities and used to deduce reaction mechanisms[3,4]. Infrared spectroscopy (IR) involves the interaction of infrared radiation (usually mid-IR within the wavenumber range of 400 – 4000 cm^{-1}) with the substrate. IR radiation is able to excite to quantum transitions of electrons in the substrate among vibrational levels if the energy differences between these levels match the energy of photons in the IR beam, thus generating fingerprint spectra of these vibrational modes[5]. In order for a vibrational mode to be IR active, the mode must be associated with changes in the dipole moment. In contrast to IR, Raman spectroscopy is based on the inelastic scattering of photons from sample surface. Typically, a source of monochromatic light (usually a laser in the visible, near infrared, or ultraviolet range) is shined on the sample. Photons excite the substrate into an excited energy state, and photons are emitted when the substrate returns to the ground state. Scattered photons with energy distinct from the incident light contain information regarding the vibration levels of the substrate, and are used to generate Raman spectra. For a vibrational mode to be Raman active, the mode must be associated with a change in the polarizability.

Complementarity of IR and Raman Spectroscopies. IR and Raman spectroscopies are generally recognized as complementary vibrational techniques for probing molecular structure. For example,

the symmetric stretching vibration of a homo-diatomic molecule does not have a permanent dipole moment due to its symmetry, which makes this mode IR inactive. In contrast, the polarizability of a homo-diatomic molecule is expected to change along the normal coordinate in the stretching mode due to the displacement of nuclei, making the vibration mode Raman active. For antisymmetric stretching or bending vibrations, the dipole moment changes sign, and thus the corresponding modes are IR active. Although the changes of the polarizability in such modes are also non-zero, they are symmetrical upon inversion of the sign of the reaction coordinate, leading to an approximately harmonic changes of the polarizability for small displacements. Therefore, both the asymmetric stretching and bending vibrations are typically Raman inactive. In principle, as for large molecules, the dissection of bond dipoles and bond polarizabilities can be conducted in a similar conceptual approach. For modes that are both IR and Raman active of a molecular species, the IR and Raman peaks are expected to appear at the same wavenumber[6].

Challenges and Significance of Combining Surface Enhanced IR and Raman Spectroscopies. Although IR and Raman spectroscopies are generally considered complementary, they are seldomly used together when investigating electrochemical reactions. The utilization of IR spectroscopy in aqueous solutions is challenging due to the intense absorption of water, leading to to severe loss of spectral signal with any significant light path through water. In order to minimize the path length of the IR light through aqueous electrolytes, two different configurations of infrared reflection absorption spectroscopy (IRAS) and attenuated total reflection (ATR) were developed, which are applicable to a wide range of surfaces, including well-defined single-crystal facets[5]. In contrast to surface enhanced IR spectroscopy, surface enhanced Raman spectrscopy is limited to a relatively few substrates with surface plasmonic effects, such as Au, Ag, and Cu, and requires

sufficient surface roughness due to the low intensity of scattering light[7,8]. Therefore, the electrode surface structures, experimental setups and conditions are usually different between IR and Raman experiments.

In electrochemical catalysis, reactive molecules are expected to adsorb on catalyst surface prior to the subsequent bond activations, which could significantly alter their vibrational modes compared to those in their free molecular states and lead to shifts of vibrational frequencies[9]. For surface adsorbed molecules, variations in adsorption sites, such as atop, bridge, and hollow sites, could induce to variations in adsorption energies and configurations, in turn leading to different reaction rates[10]. These different adsorption configurations and surface microenvironments could lead to varied dipole moments and polarizabilities. Any given technique has detection biases that favor certain types of intermediates, thus the correlation between the reaction performance and species detected by a single technique could be incomplete. In particular, the possibility of adsorbates solely responsible for catalytic activities being (largely) inactive with IR or Raman spectroscopy cannot be ruled out. This highlights the importance of combining multiple characterization techniques to provide a more complete understanding of catalytic systems.

Supplementary Figures

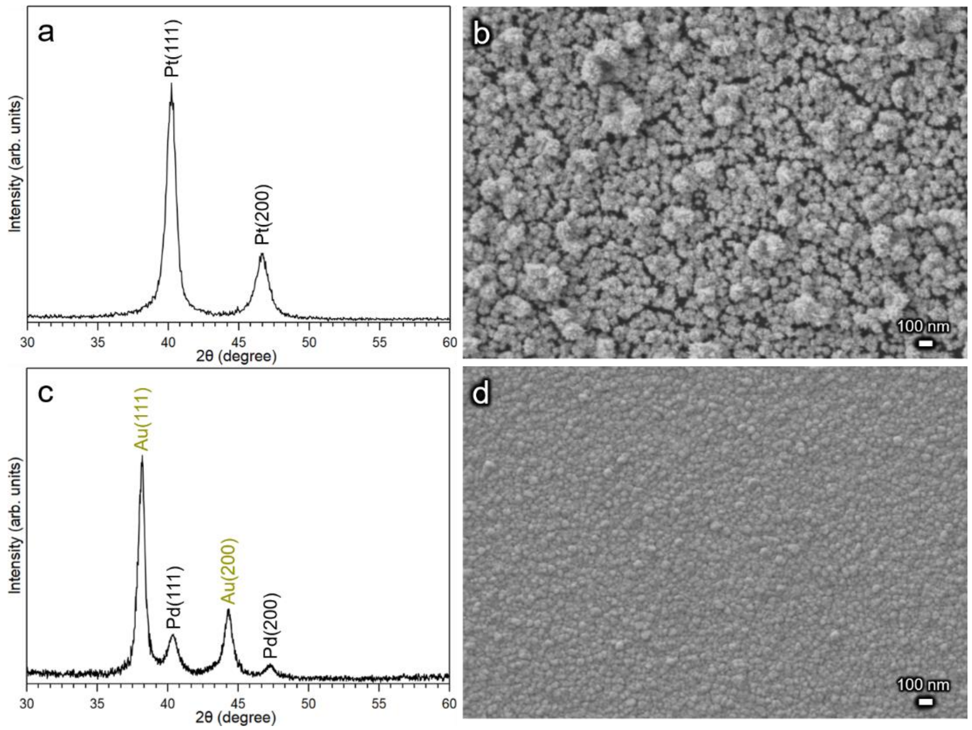

Supplementary Figure 1 Physical characterizations of Pt and Pd flims. XRD patterns and SEM images of (a and b) Pt film and (c and d) Pd film on gold substrate layer.

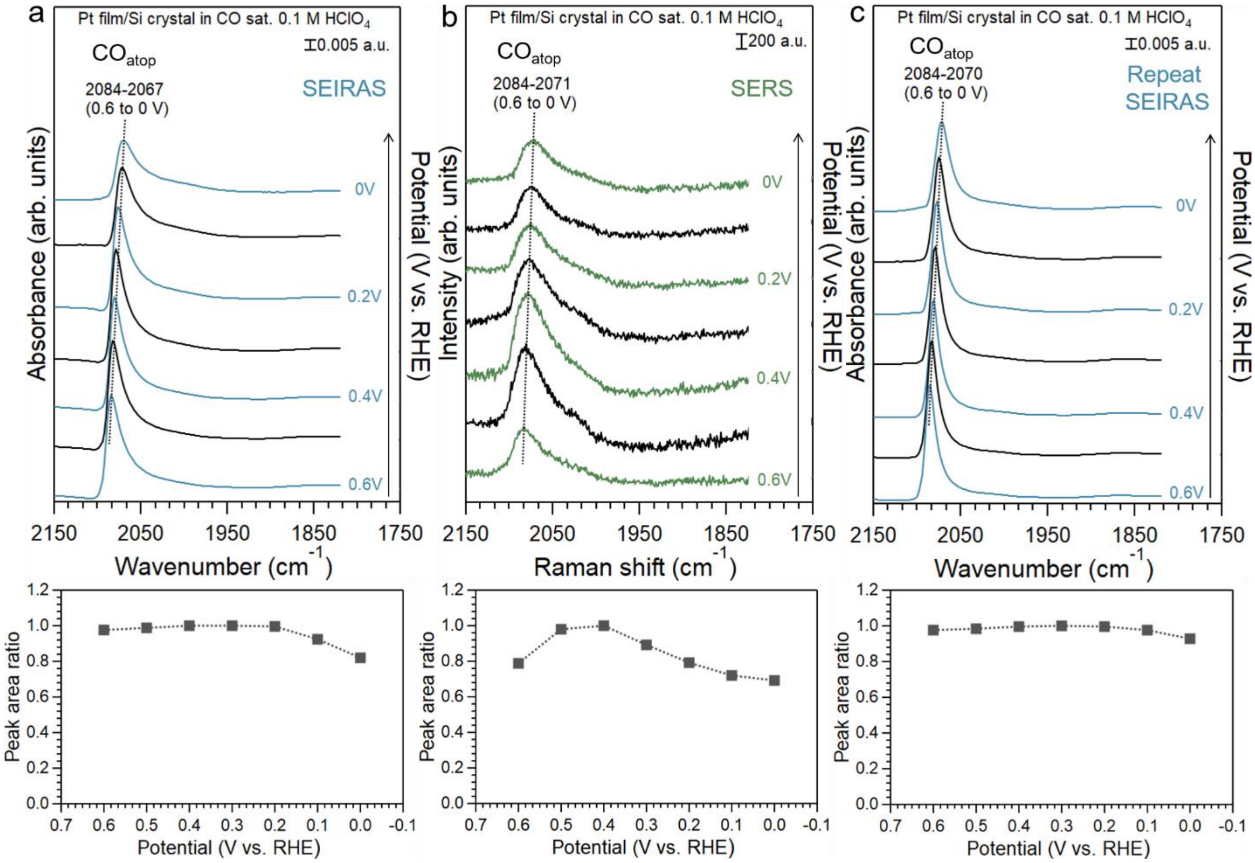

Supplementary Figure 2 Tandem in-situ SEIRAS and SERS tests on Pt film. (a) In-situ SEIRA spectra on polycrystalline Pt film in CO saturated 0.1 M $HClO_4$ (pH 1.2) and the normalized peak area of CO band as a function of potential. The background was collected at 0.6 V under Ar purge. (b) Tandem in-situ SER spectra on the same Pt film under identical conditions as those in (a) and the normalized peak area of CO band as a function of potential. (c) The repeat of in-situ SEIRA spectra on the same Pt film after SERS test under identical conditions as those in (a) and the normalized peak area of CO band as a function of potential.

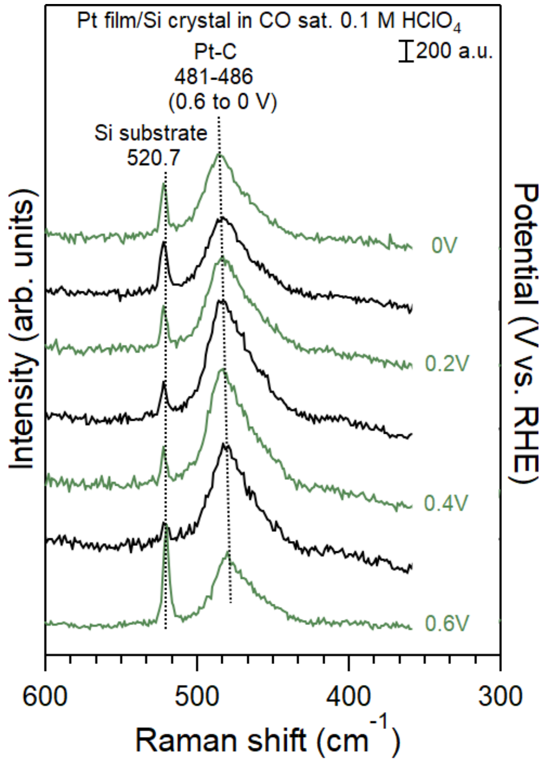

Supplementary Figure 3 SER spectra on Pt flim. The low wavenumber region of in-situ SER spectra on polycrystalline Pt film in CO saturated 0.1 M $HClO_4$ (1.2).

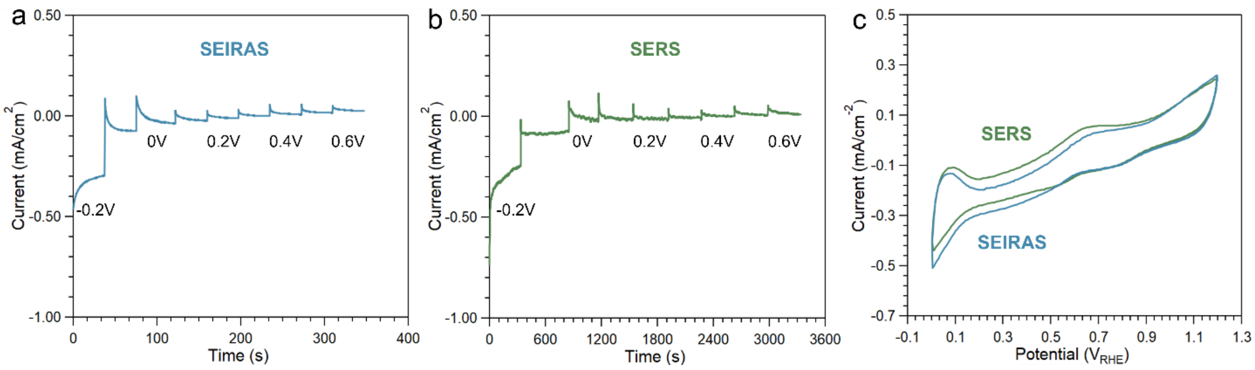

Supplementary Figure 4 The current densities and CV curves on Pt flim. Current density during the in-situ (a) SEIRAS and (b) SERS tests on polycrystalline Pt film in CO sat. 0.1 M $HClO_4$. (c) Cyclic voltammetry curves on the same Pt film in Ar sat. 0.1 M $HClO_4$ using SEIRAS (blue) and SERS (green) cells, respectively.

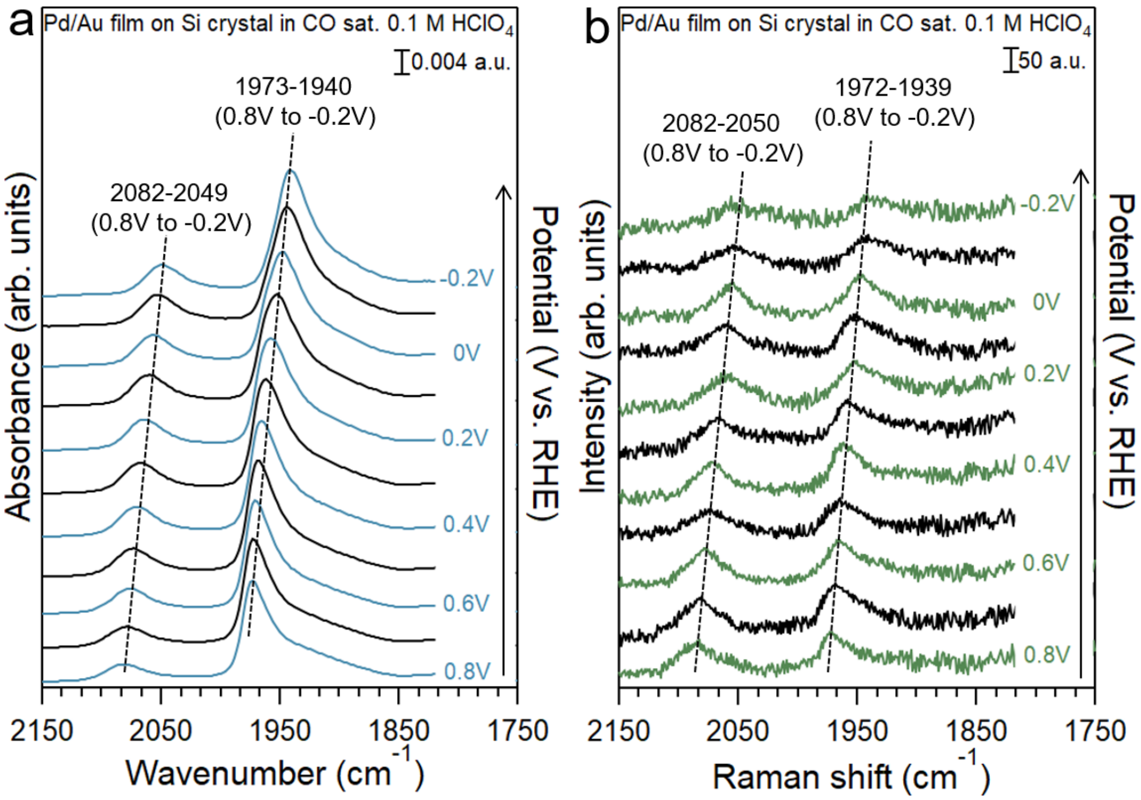

Supplementary Figure 5 Tandem in-situ SEIRAS and SERS tests on Pd film. (a) In-situ SEIRA spectra on polycrystalline Pd film in CO sat. 0.1 M $HClO_4$ (pH 1.2). The background was collected at -0.2 V under Ar purge. (b) Tandem in-situ SER spectra of CO adsorption on the same Pd film under identical conditions as those in (a). The absence of CO peak on gold, whose frequency is over 2100 cm^{-1} under 0.6 V[1], indicates the complete coverage of gold substrate by Pd film.

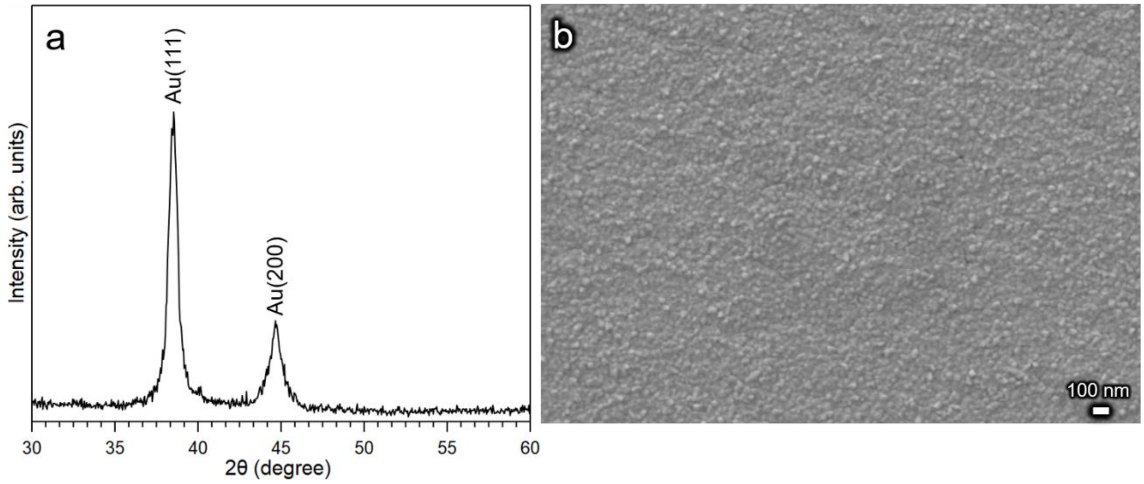

Supplementary Figure 6 Physical characterizations of Au film. (a) XRD patterns and (b) SEM image of Au film.

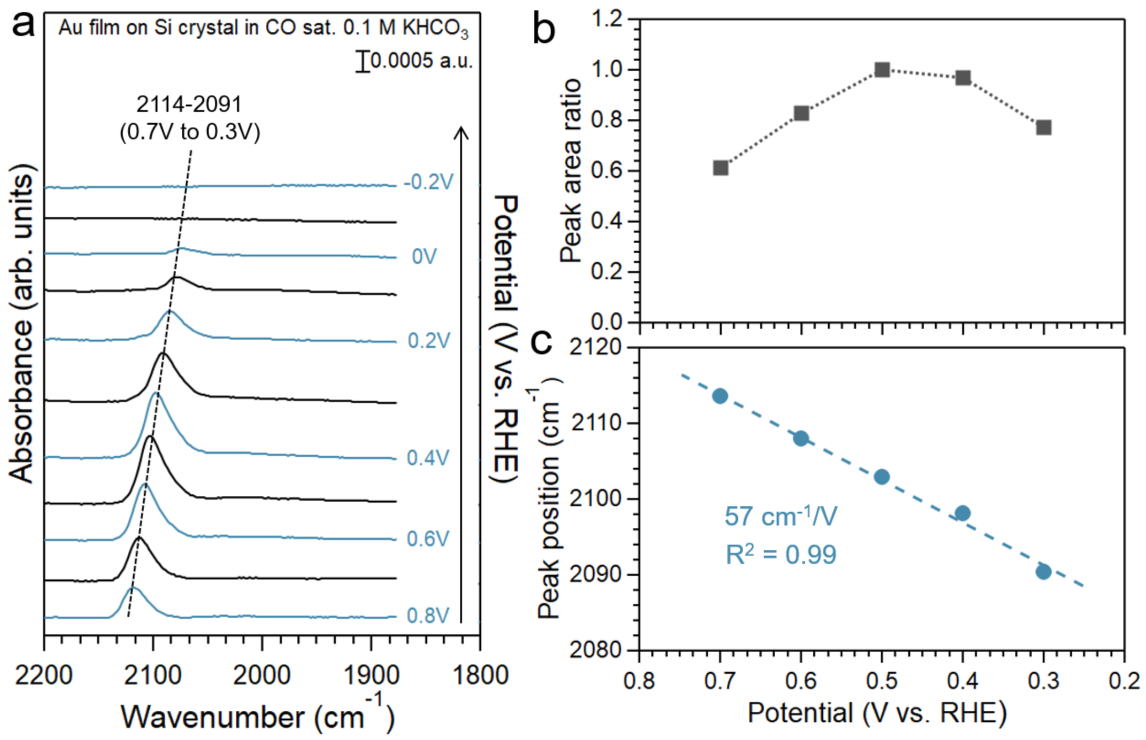

Supplementary Figure 7 In-situ SEIRAS test on Au film. (a) In-situ SEIRA spectra on polycrystalline Au film in CO saturated 0.1 M $KHCO_3$ (pH 8.9). The background was collected at -0.4 V under Ar purge. (b) The normalized peak area of the CO adsorption bands as a function of potential in the range of 0.7 to 0.3 V. (c) The potential dependence of CO band frequency. Stark tuning rate is determined through the linear fits of the point data between 0.7 and 0.3 V, at which the peak area is greater than 60% of the maximum.

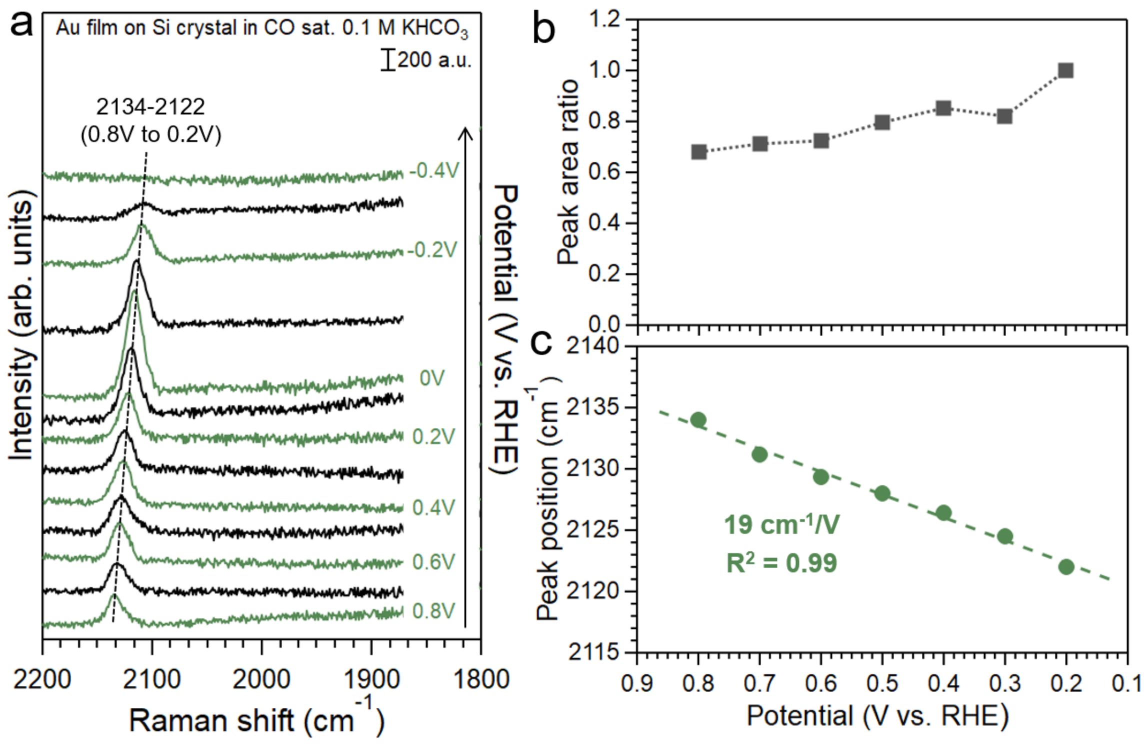

Supplementary Figure 8 In-situ SERS test on Au film. (a) Tandem in-situ SER spectra on the same Au film under identical conditions as those in supplementary Figure 7. (b) The normalized peak area of the CO adsorption bands as a function of potential in the range of 0.8 to 0.2 V. (c) The potential dependence of CO band frequency. Stark tuning rate is determined through the linear fits of the point data between 0.8 and 0.2 V, at which the peak area is greater than 60% of the maximum.

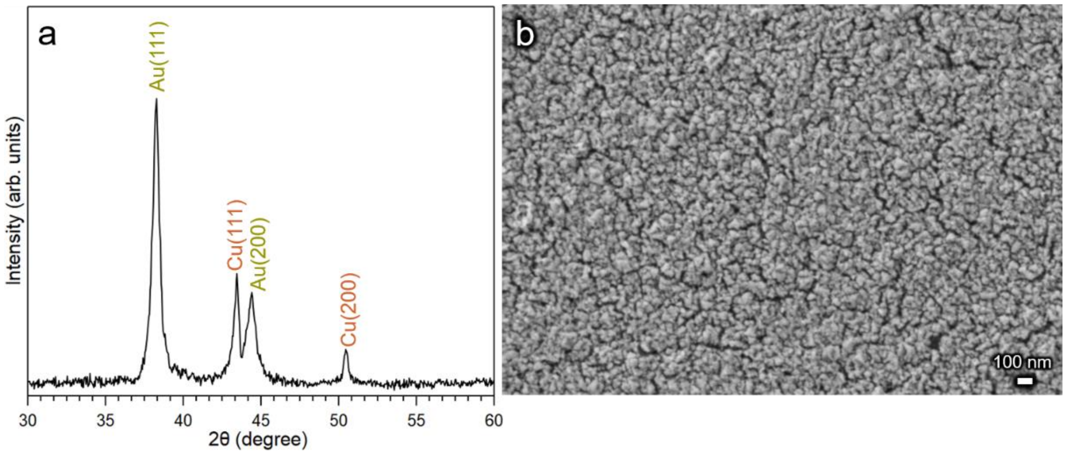

Supplementary Figure 9 Physical characterizations of OD-Cu film. (a) XRD patterns and (b) SEM image of OD-Cu film on gold substrate layer. The absence of Cu_2O characteristic peaks in XRD patterns indicates the complete reduction of it into OD-Cu.

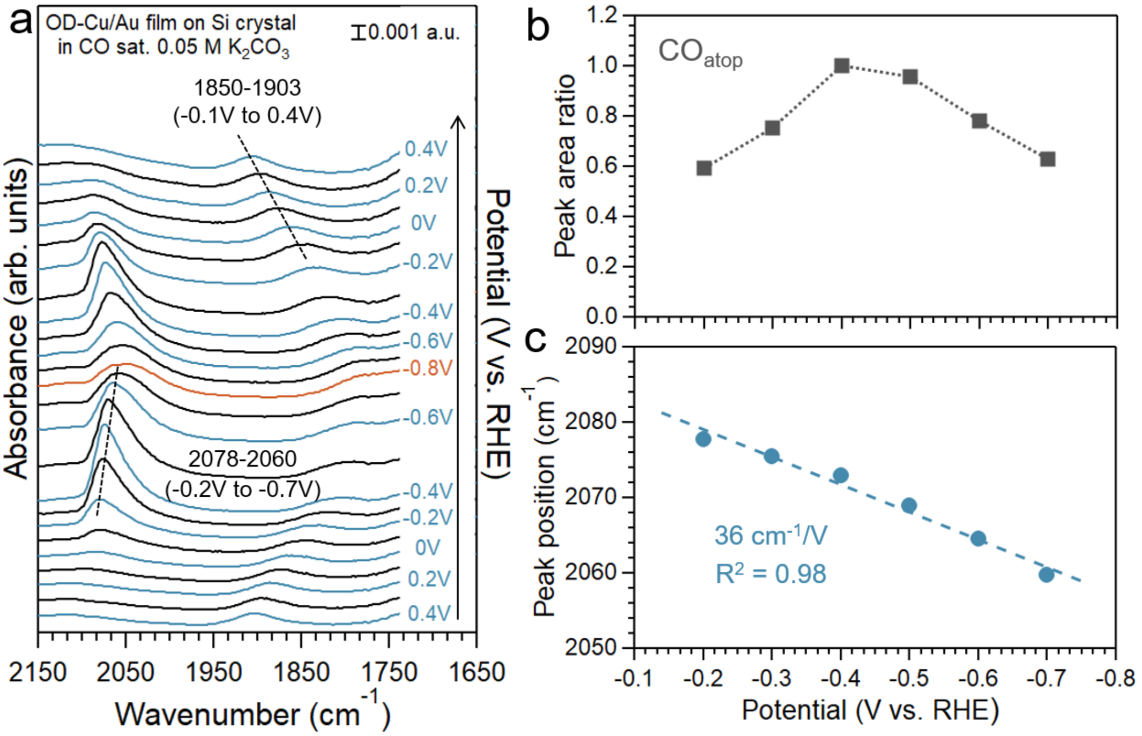

Supplementary Figure 10 In-situ SEIRAS test on OD-Cu film. (a) In-situ SEIRA spectra on polycrystalline OD-Cu film in CO sat. 0.05 M K_2CO_3 (pH 10.6). The background was collected at 0.1 V under Ar purge. (b) The normalized peak area of the CO_{atop} adsorption bands as a function of potential in the range of -0.2 to -0.7 V. (c) The potential dependence of CO band frequency. Stark tuning rate is determined through the linear fits of the point data between -0.2 and -0.7 V, at which the peak area is greater than 60% of the maximum.

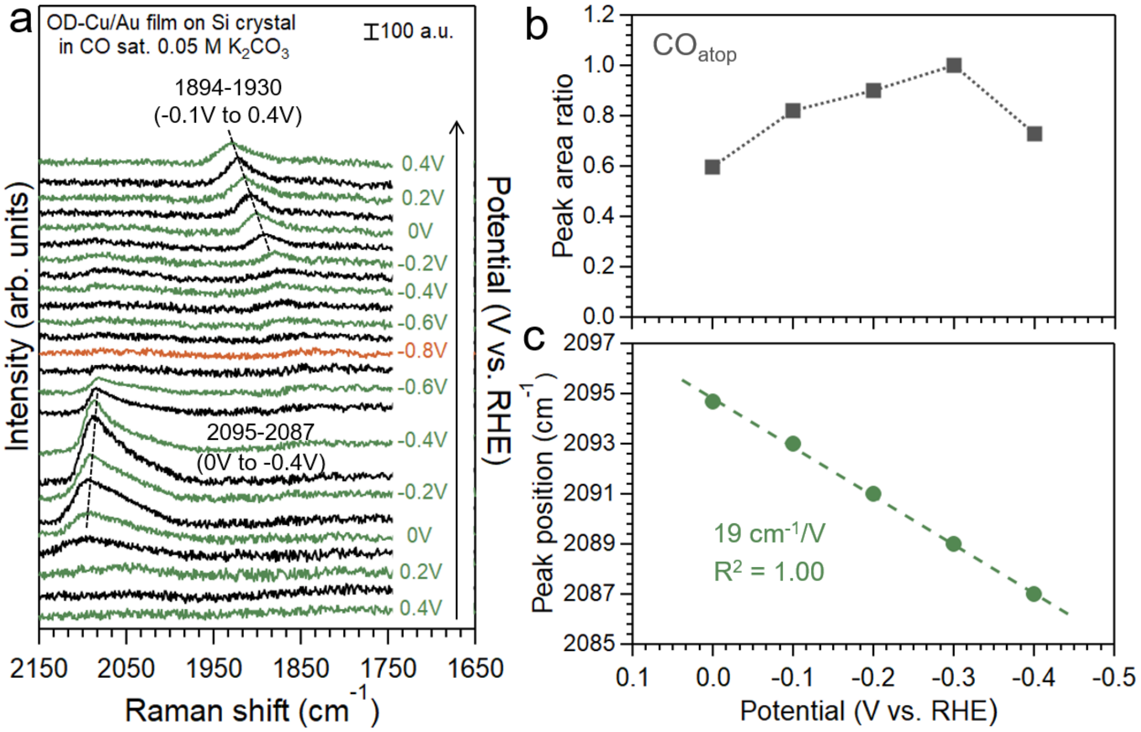

Supplementary Figure 11 In-situ SERS test on OD-Cu film. (a) In-situ SER spectra on another fresh OD-Cu film and under identical conditions as those in Supplementary Figure 10. (b) The normalized peak area of the CO_{atop} adsorption bands as a function of potential in the range of 0 to -0.4 V. (c) The potential dependence of CO band frequency. Stark tuning rate is determined through the linear fits of the point data between 0 and -0.4 V, at which the peak area is greater than 60% of the maximum.

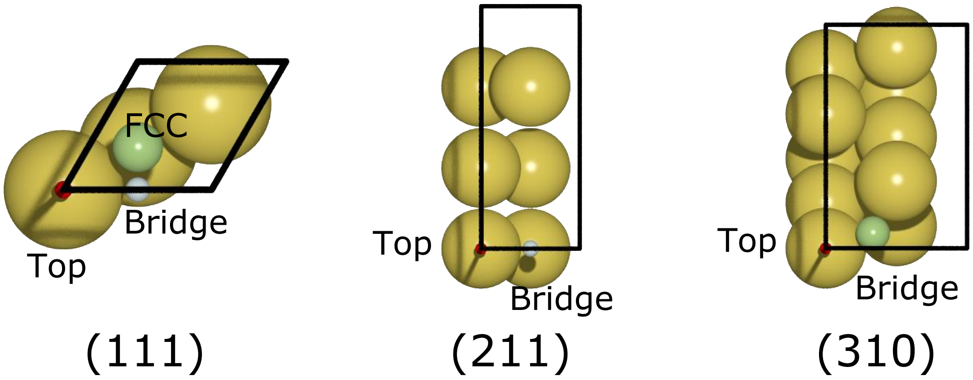

Supplementary Figure 12 Schematic of different sites. Schematic of the different sites (marked with the various generic adsorbates) used in the analysis of Figure 4 for (111), (211) and (310) surfaces.

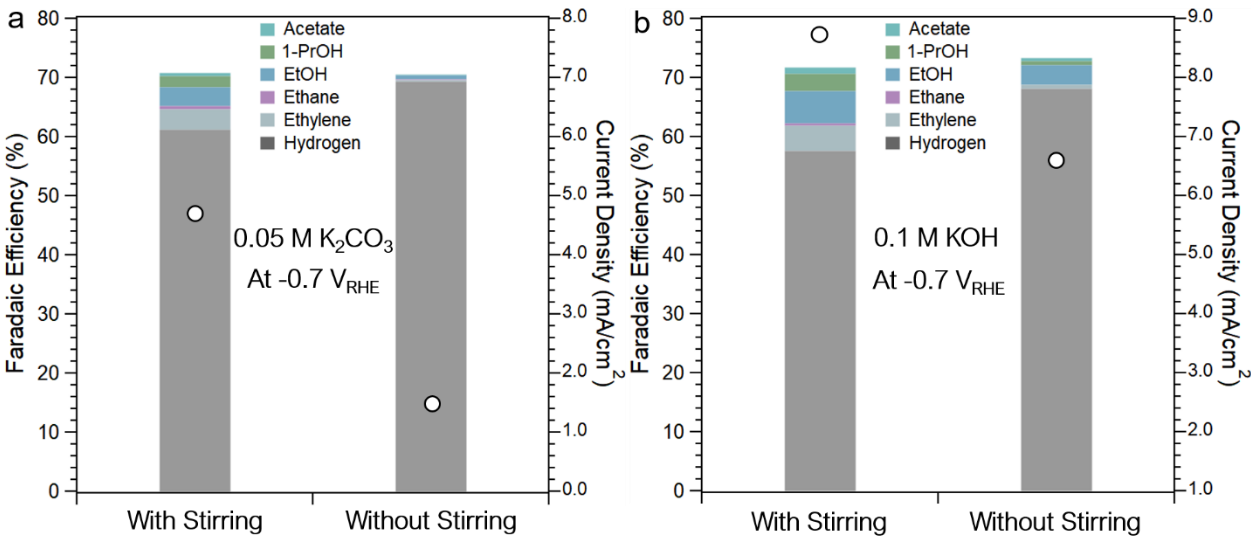

Supplementary Figure 13 CORR activity test on OD-Cu film. Faradaic efficiency of products formed along with the CORR current density (hollow circle) with and without stirring on OD-Cu film at -0.7 V_{RHE} in CO saturated (a) 0.05 M K_2CO_3 and (b) 0.1 M KOH. The reaction was conducted in the SEIRAS spectrochemical cell (Figure 1a).

Supplementary References

1. Marco Dunwell, Q. L., Jeffrey M. Heyes, Jonathan Rosen, Jingguang G. Chen, Yushan Yan, Feng Jiao, and Bingjun Xu. The Central Role of Bicarbonate in the Electrochemical Reduction of Carbon Dioxide on Gold. *J. Am. Chem. Soc.* **139**, 3774-3783 (2017).
2. Le Ru, E. C. & Etchegoin, P. G. Single-molecule surface-enhanced Raman spectroscopy. *Annu. Rev. Phys. Chem.* **63**, 65-87 (2012).
3. Malkani, A. S., Dunwell, M. & Xu, B. Operando Spectroscopic Investigations of Copper and Oxide-Derived Copper Catalysts for Electrochemical CO Reduction. *ACS Catal.* **9**, 474-478 (2018).
4. Jiang, S., Klingan, K., Pasquini, C. & Dau, H. New aspects of operando Raman spectroscopy applied to electrochemical CO_2 reduction on Cu foams. *J. Chem. Phys.* **150**, 041718 (2019).
5. Kas, R. *et al.* In-Situ Infrared Spectroscopy Applied to the Study of the Electrocatalytic Reduction of CO_2: Theory, Practice and Challenges. *Chemphyschem* **20**, 2904-2925 (2019).
6. McQuarrie, D. & Simon, J. Physical Chemistry: A Molecular Approach. in *University Science Books: VA*, (1997).
7. Linic, S., Christopher, P. & Ingram, D. B. Plasmonic-metal nanostructures for efficient conversion of solar to chemical energy. *Nat. Mater.* **10**, 911-921 (2011).
8. Stiles, P. L., Dieringer, J. A., Shah, N. C. & Van Duyne, R. P. Surface-enhanced Raman spectroscopy. *Annu. Rev. Anal. Chem.* **1**, 601-626 (2008).
9. Roth, J. D. & Weaver, M. J. Potential-difference surface infrared spectroscopy under forced hydrodynamic flow conditions: control and elimination of adsorbate solution-phase interferences. *Anal. Chem.* **63**, 1603-1606 (1991).
10. Gunathunge, C. M., Ovalle, V. J., Li, Y., Janik, M. J. & Waegele, M. M. Existence of an Electrochemically Inert CO Population on Cu Electrodes in Alkaline pH. *ACS Catal.* **8**, 7507-7516 (2018).
